# A deep convolutional neural network-based automatic delineation strategy for multiple brain metastases stereotactic radiosurgery

**DOI:** 10.1371/journal.pone.0185844

**Published:** 2017-10-06

**Authors:** Yan Liu, Strahinja Stojadinovic, Brian Hrycushko, Zabi Wardak, Steven Lau, Weiguo Lu, Yulong Yan, Steve B. Jiang, Xin Zhen, Robert Timmerman, Lucien Nedzi, Xuejun Gu

**Affiliations:** 1 School of Electrical Engineering and Information, Sichuan University, Chengdu, Sichuan, China; 2 Department of Radiation Oncology, The University of Texas Southwestern Medical Center, Dallas, TX, United States of America; 3 Department of Biomedical Engineering, Southern Medical University, Guangzhou, Guangdong, China; North Shore Long Island Jewish Health System, UNITED STATES

## Abstract

Accurate and automatic brain metastases target delineation is a key step for efficient and effective stereotactic radiosurgery (SRS) treatment planning. In this work, we developed a deep learning convolutional neural network (CNN) algorithm for segmenting brain metastases on contrast-enhanced T1-weighted magnetic resonance imaging (MRI) datasets. We integrated the CNN-based algorithm into an automatic brain metastases segmentation workflow and validated on both Multimodal Brain Tumor Image Segmentation challenge (BRATS) data and clinical patients' data. Validation on BRATS data yielded average DICE coefficients (DCs) of 0.75±0.07 in the tumor core and 0.81±0.04 in the enhancing tumor, which outperformed most techniques in the 2015 BRATS challenge. Segmentation results of patient cases showed an average of DCs 0.67±0.03 and achieved an area under the receiver operating characteristic curve of 0.98±0.01. The developed automatic segmentation strategy surpasses current benchmark levels and offers a promising tool for SRS treatment planning for multiple brain metastases.

## Introduction

The incidence of brain metastases has increased with the advanced modern cancer therapy technology and prolonged cancer survival. [1]. Stereotactic radiosurgery (SRS), a standard of care for brain metastases [2], requires accurate delineation of tumor/target volumes for treatment planning, but manually contouring multiple brain metastases can be a time- and labor-intensive process. Developing an accurate and efficient automated delineation tool would benefit clinical practice by improving the efficiency of SRS treatment planning.

Researchers have been investigating automatic brain tumor segmentation methods for decades [3,4] and have developed various tools [5–7]. Currently available automatic brain tumor delineation methods can be divided into two categories: information theory-based methods and learning-based methods [3]. The information theory-based methods use image data itself and utilize traditional image processing tools to detect abnormalities. Exemplary algorithms include watershed segmentation algorithms, active contour algorithms, and region-growing segmentation algorithms [8–11]. Learning-based methods consider segmentation tasks as classification problems and require a certain number of expert-segmented images to train classification models. On those expert-segmented images, manually-designed image features, such as mean, standard deviation, gray level co-occurrence matrix (GLCM), and local binary pattern features (LBP), are extracted and fed into machine learning models, such as support vector machine (SVM) or artificial neural network (ANN), to classify target abnormalities [12–17].

Automatic brain metastases segmentation requires special considerations in its clinical implementation. First, SRS is often used to treat small tumors, e.g. the tumor diameter is less than 1cm [2], which are easy to miss in information theory-based segment methods. Second, clusters of brain metastases complicate automatic segmentation, and simultaneous delineation of multiple lesions is difficult. Third, contrast-enhanced T1 magnetic resonance imaging (MRI) is generally the only imaging modality acquired for treatment planning, which eliminates the application of many advanced multi-modality image segmentation tools. Furthermore, SRS is usually a one-day outpatient procedure, which requires fast segmentation for a rapid clinical workflow.

Recently, we developed an automatic brain metastatic tumor segmentation strategy for an SRS clinical workflow [7]. The developed strategy integrates a set of traditional image processing tools and takes advantage of image intensity information to discriminate tumor regions from surrounding tissues. This method achieved high accuracy in automatic contouring in both simulated data and clinical patient image sets. However, the developed method has difficulties in segmenting small brain metastases with volumes less than 1.500cc, especially when the tumor is surrounded by other high intensity structures, such as the superior sagittal sinus or a confluence of sinuses. Its intensity histogram-based abnormalities-detecting strategy hinders its application in segmenting small-size tumors, since a limited number of voxels in a small-size has an undistinguishable intensity histogram from surrounding structures. Conventional artifact feature-based learning methods could fail with small-size tumor segmentation as well, because the limited number of voxels is unlikely to provide statistically significant features for segmentation. Deep learning convolutional neural networks (CNN), which require neither manual image feature extraction nor tumor intensity histogram, may have advantages in classifying small-size abnormalities. Advantage in classifying small targets have been proved in reference [18] [19]. The CNN algorithm utilizes a stack of sequentially connected convolutional filters to study the nonlinear relationship between abnormal voxels and their neighbors, and automatically derives a voxel characterization model. This self-learning procedure is promising for small-size tumor segmentation.

In this paper, we report our newly developed CNN-based brain metastases auto-segmentation strategy. This work aims to delineate the small lesions (<1.5cc) accurately and efficiently. The network architecture is branchy and made up of three sub-paths to incorporate multi-scale information to perform delineation of small lesions on MRI.

## Methods and materials

### Ethics statement

This retrospective patient study was approved by Human Research Protection Program Office (HRPPO)/Institutional Review Board (IRB) of The University of Texas Southwestern Medical Center. All methods in this study were conducted in accordance with the relevant guidelines and regulations. Considering that this is not a therapeutical treatment study, our institutional review board waived the need for obtaining written informed consent from the participants.

### Automatic delineation workflow

**Fig 1:**
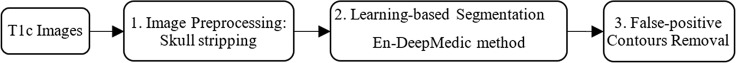
Workflow of the proposed deep learning-based brain metastases auto-segmentation.

The auto-segmentation workflow that we developed is illustrated in Fig 1. The entire workflow can be divided into three sections: image preprocessing, segmentation, and false positive contour removal. The first section, image preprocessing, removes the skull from the original T1c image by employing a robust learning-based MRI brain extraction system (ROBEX) [20]. ROBEX combines a discriminative and a generative model to achieve the final result. When a new image is presented to ROBEX, the tool uses a Random Forest classifier to detect the brain boundary. Then the generative model is explored to find a highest likelihood contour. The brain contour is refined by free deformation and used for skull stripping. The third section removes false positive contours by utilizing the spherical geometry characteristics of brain metastases. Essentially, we use a sphericity metric [21] to quantify the delineated structure shape. The structure with a sphericity value smaller than a predefined threshold is removed from the final contour sets. The intermediate second step is a learning-based segmentation method consisting of a CNN architecture, which we will describe in detail in the following sections.

### A Modified DeepMedic CNN architecture

**Fig 2:**
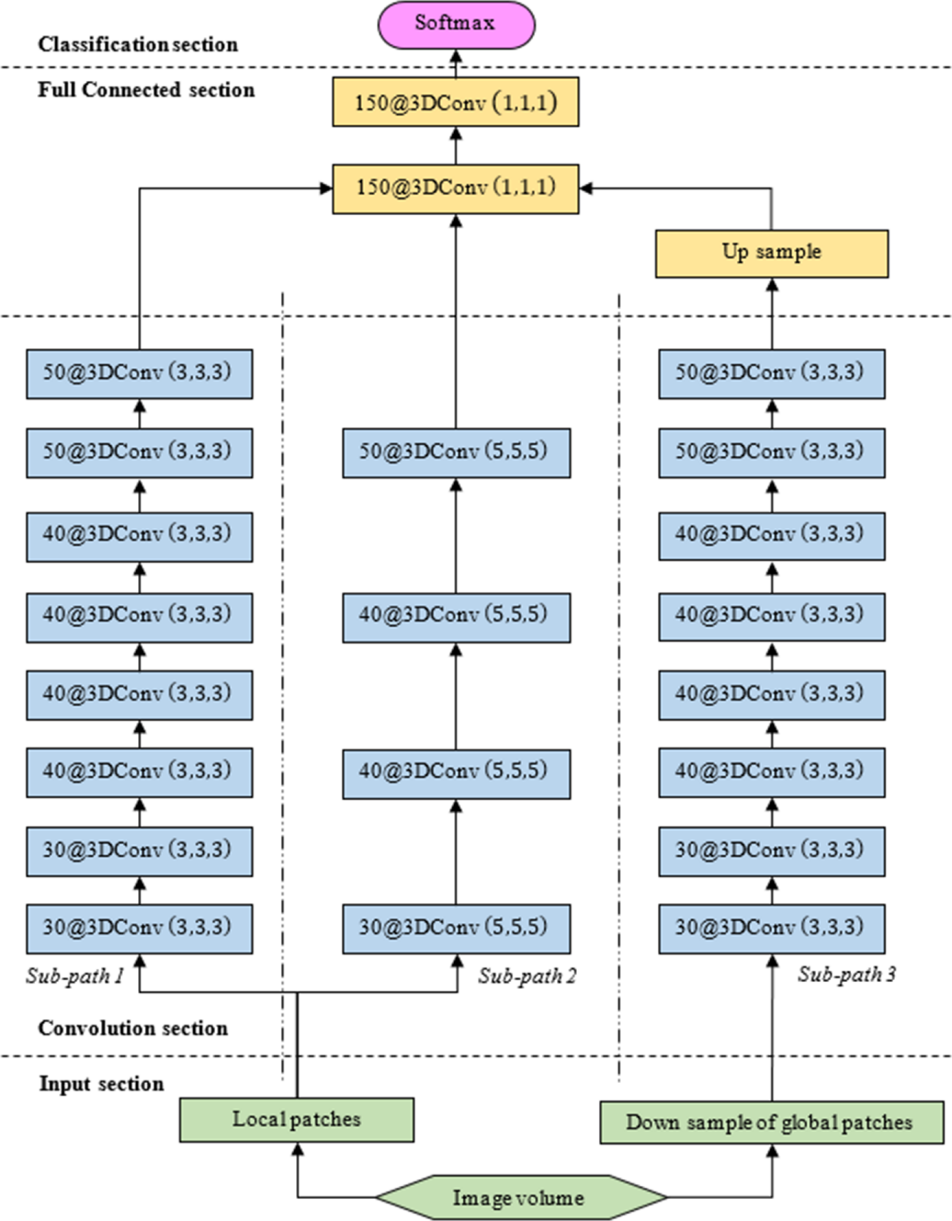
En-DeepMedic architecture. ***α***^***l***^@3DConv (**k**_**1**_,**k**_**2**_,**k**_**3**_): ***α***^***l***^ is the number of feature maps captured at layer *l*, (**k**_**1**_,**k**_**2**_,**k**_**3**_) is convolution filters size.

A CNN framework treats segmentation as a voxel-wise classification problem. The lesion can be delineated from the background if the probability of each image voxel belonging to the target is known. Our work was inspired by DeepMedic, a CNN architecture proposed by Kamnitsas *et al*. [22]. The network architecture consists of a sequence of four sections: input, convolution, fully connected, and classification sections, as illustrated in Fig 2. The input section processes the original images to obtain the designed image patches. The designed image patches are then fed into a convolution section, where multi-layer convolutional filters operate and output feature maps. The convolution section is followed by a fully connected layer that groups all feature maps. The final classification section calculates a prediction score to classify each image voxel and yield a segmentation map. Our proposed CNN method enhances the original DeepMedic structure by including an additional sub-path (sub-path 2) to capture multi-scale image features for accurate image segmentation. In addition, the entire structure is implemented on a graphic processing unit (GPU) platform to improve computation efficiency. For simplicity, we call this modified DeepMedic CNN architecture “En-DeepMedic” in this paper.

#### Input section

The input section generates image patches for the rest of the network. En-DeepMedic is a voxel-wise classification system, where each voxel is classified based on the linear and non-linear relationship between the focal voxel’s intensity and its neighbors. Because of the large image size, as the case of the 3D brain MRI images studies in the paper, calculating the linear or nonlinear relationship between all voxels in the entire image is computation-intensive. We divide the image into small patches to calculate the voxel relationship within a limited region instead of the entire image. Using small image patches saves computational time and memory space. Such strategy is important for GPU implementation; especially on-chip memory is limited on the current market-available GPU cards.

**Fig 3:**
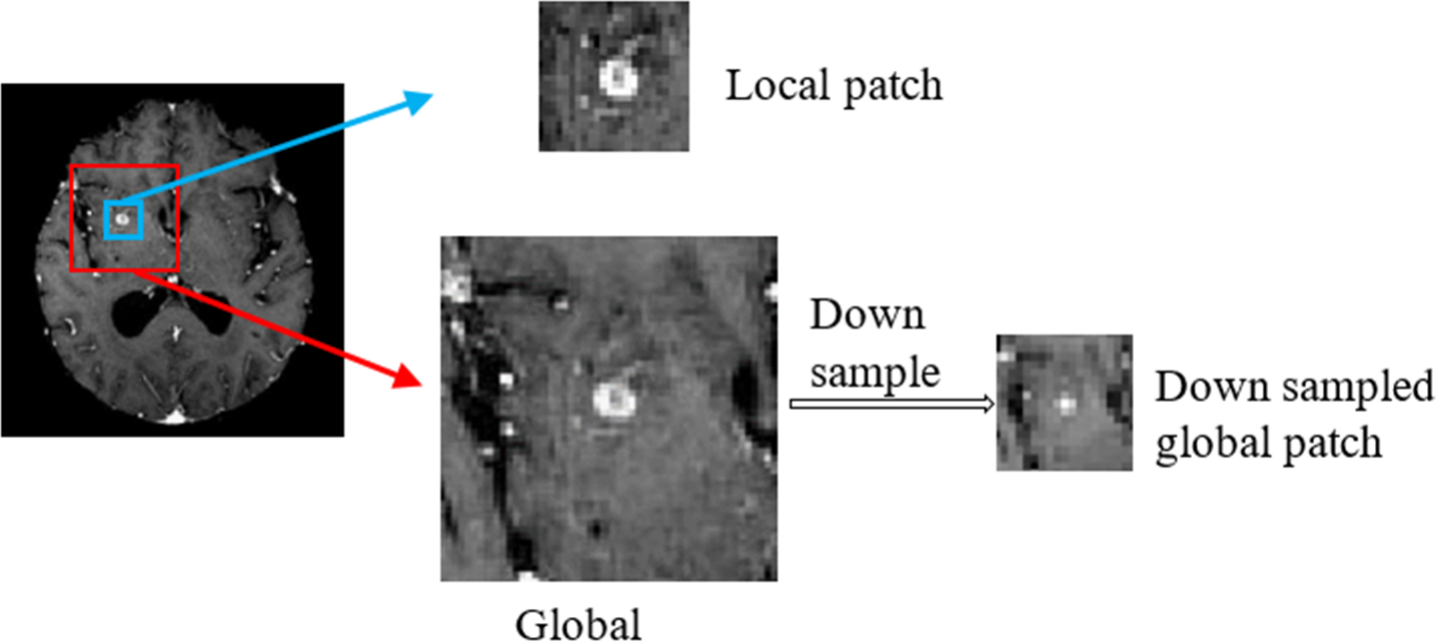
Illustration of image patch extraction.

The En-DeepMedic architecture extracts both local and global patches as inputs for the convolution section. Fig 3 illustrates a two-dimensional patch extraction strategy, though the actual image patches used in the algorithm are three-dimensional. Each extraction selected the central voxel at random and simultaneously extracted concentric local and global image patches. Neighboring voxels around the central voxels form the local patch, which provides local information. The global patch covers a larger region, which contains relative global information. To mitigate the computational burden caused by the larger global patch, we down-sampled all global patches. In this study, we specified the local image patch size as (25, 25, 25) and down-sampled the global patches from (57, 57, 57) to (19, 19, 19).

#### Modified convolution section

The convolution section has multiple layers that sequentially capture the image features by convolution operations. These captured image features include low-level features, such as edge and corner, and high-level features, such as non-linear intensity relationship between neighboring voxels. The feature maps are the output of each convolution layer. The entire convolution section comprises three different sub-paths. Sub-paths 1 and 2 use the same local patch with different filter sizes to capture different neighborhood patterns, while sub-path 3 works on the down-sampled global patch to reveal global features. For each sub-path, let *L* be the depth of the convolution filter stack, which is the number of convolution layers used in a sub-path. *α*^*l*^ (*l* ∈ [1,*L*]) be the number of feature maps in the *l*^*th*^ layer, and $F_{i}^{l}$ (*i* ∈ [1,*α*^*l*^]) be the *i*^*th*^ feature map of the *l*^*th*^ layer. In this cascaded network, $F_{i}^{l}$ is calculated by nonlinearly activating the convolution of the *l* − 1^*th*^ layer feature map and the *l*^*th*^ layer filter with certain bias, as shown in Eq 1:

$$F_{i}^{l}=g\left(\sum_{j=1}^{\alpha^{l-1}}F_{j}^{l-1}*W_{ji}^{l}+b_{i}^{l}\right).$$ (1)

**Fig 4:**
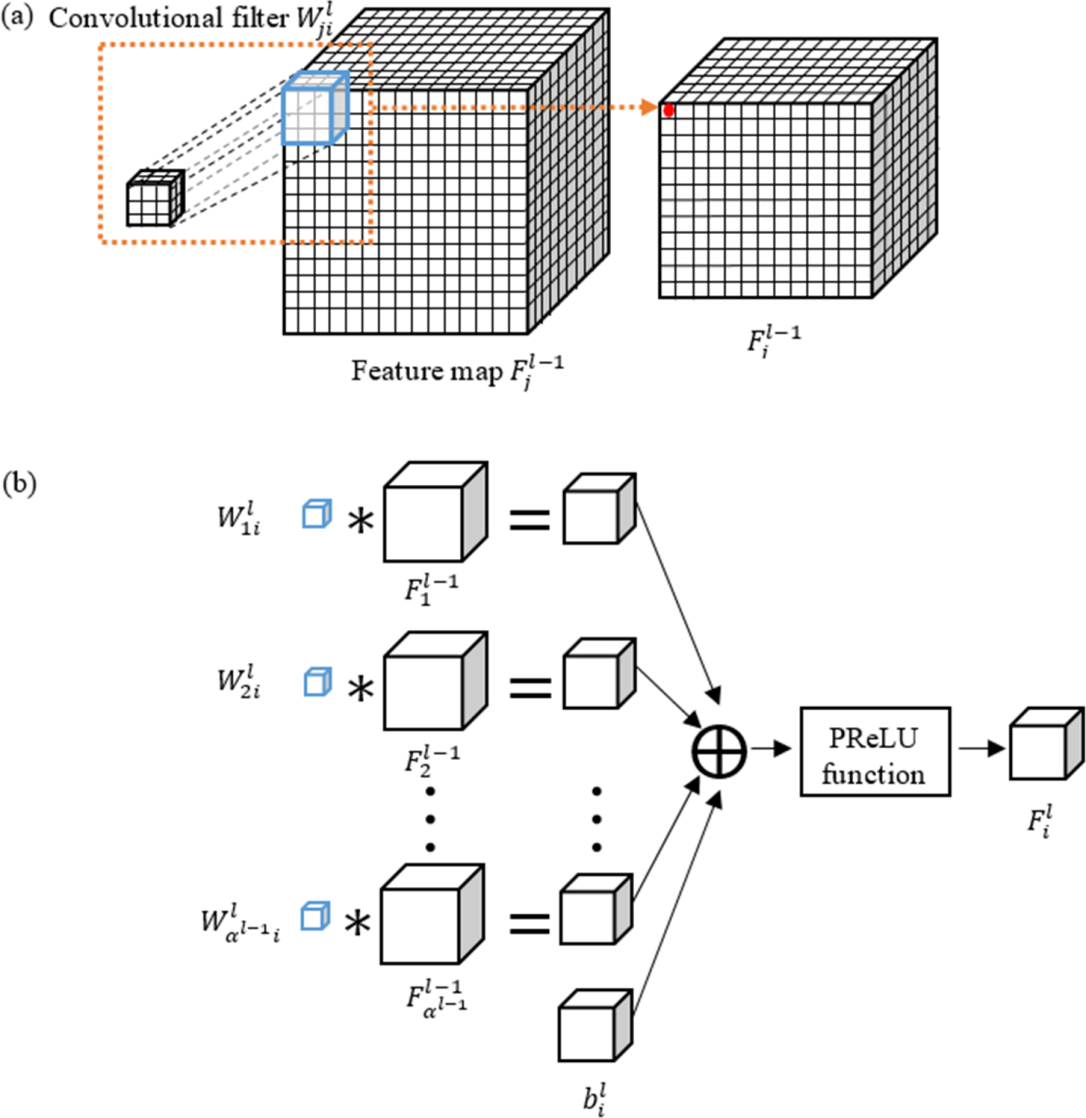
Process of 3D convolution layer. (a) 3D convolution of a feature map with a filter. (b) Generation of the *i*th feature map in the *l*th layer.

Here, *g*(∙) is the PReLU function [23]. The PReLU is a neuron activation function defined as $g(x)=\left\{ \begin{array}{c} x,x>0\\ ax,x\leq 0 \end{array}\right.$, where a is a network parameter. $W_{ji}^{l}$ is the filter connecting the *j*^*th*^ feature map in the *l* − 1^*th*^ layer and the *i*^*th*^ feature map in the *l*^*th*^ layer, and $b_{i}^{l}$ is the bias from the artificial neuron model [24]. A typical 3D convolutional operation is illustrated in Fig 4(A). The generation of the *i*^*th*^ feature map in the *l*^*th*^ layer is illustrated in Fig 4(B). The filter number of layer *l* is *α*^*l*−1^ ∙ *α*^*l*^. The total filter coefficient number of each individual convolutional layer stack is $N=\sum_{l=1}^{L}\alpha^{l-1}∙\alpha^{l}∙k_{1}^{l}∙k_{2}^{l}∙k_{3}^{l}$, where $\left(k_{1}^{l},k_{2}^{l},k_{3}^{l}\right)$ is the filter size of the *l*^*th*^ layer. As there are 3 sub-paths in this section, the total number of to-be-learned filter coefficients is ∑_*p*_*N*_*p*_, *p* = 1,2,3. With a filter size of (3,3,3) in sub-path 1 (and 3) and (5,5,5) in sub-path 2, a local patch size of (25,25,25), and a down-sampled global patch size of (19, 19, 19), the size of the output feature map is (9,9,9) in sub-path 1 and 2 and (3,3,3) in subpath 3.

Compared to the original DeepMedic method, our En-DeepMedic is distinguished by an additional sub-path (sub-path 2) included in the convolution section. Sub-path 2 uses a larger convolution filter (5, 5, 5) and operates on every other convolution layer, unlike sub-path 1. This design is useful for capturing different multiscale features in addition to those captured by sub-path 1.

#### Fully connected section

The fully connected (FC) section fuses all of the feature maps generated by the convolution section to preserve the consistency of spatial information. The FC section operates similarly to the convolution section, where each coefficient node works as a convolution filter with size (1,1,1). It treats the output feature maps of the three sub-paths equally, which requires feature maps to have the same size.

#### Classification section

The classification section generates the categorical probability for each voxel. Like most convolutional neural networks, a softmax function maps the feature map to categorical probabilities. A cost function (Eq 2) is defined to maximize the logarithmic likelihood between the input patch, I_*s*_, and the corresponding ground truth segmented patch, c_*s*_.

$$J_{D}(\Theta ,{Ι}_{s},c_{s})=-\frac{1}{B\cdot V}\sum_{s=1}^{B}\sum_{v=1}^{V}log(p_{c_{s}^{v}}(x^{v})),$$ (2)

where *x*^*v*^ and $c_{s}^{v}$ are the *v*^*th*^ target voxel’s position and ground truth, *v* ∈ [1,*V*]. $V=f_{1}^{L}\times f_{2}^{L}\times f_{3}^{L}$, where ${(f}_{1}^{L},f_{2}^{L},f_{3}^{L})$ is the *L*^*th*^ layer feature map size. *s* ∈ [1,*B*], where *B* is the size of a batch. $p_{c_{s}^{v}}$ is the output of the softmax function. The parameter Θ is denoted as the filter coefficients and bias, which are determined during network training through the Stochastic Gradient Descent (SGD) method[25]. Here, the network training refers to using a set of segmented images (called labelled images) to find the optimal network parameters. SGD is a stochastic approximation of the gradient descent optimization method for minimizing an objective function iteratively. In each iteration, it calculates the gradient from a subset of labelled dataset. It helps to speed up the training in a large training set.

#### Hyper parameters

Before training, we predefined a set of *hyper parameters*, which could not be learned automatically through training. The hyper parameters used in our En-DeepMedic architecture are listed in Table 1 along with their associated workflows. *Initialization* generates initial coefficients for the filter weights and bias. Instead of using random values, we employed the method used by He *et al*. [23] to generate initial coefficients. The *Dropout* technique [26] avoids overfitting by randomly discarding part of the obtained coefficients in each iteration. We performed the dropout scheme on the FC section only with a dropout rate of 0.5. *Data augmentation* balances positive and negative samples, because there is a large gap between the number of tumor and non-tumor voxels. All positive patches were flipped along the sagittal plane to increase their numbers. A *k-fold cross validation strategy* trains, validates, and tests our En-DeepMedic method. We randomly partitioned the original dataset into *k* subsets with equal size and performed the training and validation phases *k* times with one subset for validation and the other *k*-1 subsets for training. *Batch size* and *epoch*s depend on computer memory and could be modified based on the machine architecture.

**Table 1:** Hyper parameters used in this study.

	BRATS validation		clinical data validation	
	*hyper parameters*	*value*	*hyper parameters*	*value*
**Initialization**	weights and bias	He method [23]	weights and bias	He method [23]
**Dropout**	convolutional section	0	convolutional section	0
	FC section	0.5	FC section	0.5
**Data Augmentation**	flipped	along sagittal plane	flipped	along sagittal plane
**Training**	batch size	10	batch size	10
	epochs	35	epochs	15
**Validation**	batch size	48	batch size	48
**Testing**	batch size	10	batch size	10

### Validation dataset

We used two groups of data to train and validate our model. One dataset was from BRATS [27], a database for evaluating brain tumor segmentation methods. BRATS provides each patient’s T1-weighted MRI with Gadolinium contrast (T1c) and T2-weighted Fluid-Attenuated Inversion Recovery (FLAIR) images. We used this dataset to compare our automatic delineation method with other competitive algorithms. All images in the dataset were resized to 1.0mm × 1.0mm × 1.0mm after skull removal. The other dataset consisted of 240 brain metastases patients with T1c MRI scans collected at the University of Texas Southwestern Medical Center (UTSW) from 2009 to 2014 [28]. The number of brain metastases per case varies from 1 to 93 (5.679 ± 8.917 per case). The mean tumor size is 0.672 ± 1.994cc. All scans were acquired on a SIEMENS 3T MRI system prior to radiosurgery on the treatment day. The brain metastases contours drawn by neurosurgeons following the standard UTSW clinical protocol were used as ground-truth in this study. Each data group was divided into three sets: training, validation, and testing. Both training and validation sets were used in the training phase to generate the proper network coefficients. The testing set was used to evaluate the trained model. BRATS (265 cases) and clinical data (225 cases) were selected and randomly assigned to the training and validation sets to generate the network coefficients.

### Evaluation metrics

We evaluated the performance of the En-DeepMedic-based auto-delineation method with respect to both geometrical measurements of individual tumor segmentation accuracy and statistical measurements of receiver operator characteristic (ROC) curve analysis. We quantitatively evaluated the geometrical accuracy of individual tumor segmentation with 1) DICE coefficients ($DCs=\frac{2(A\cap B)}{\left(A\cup B\right)}$), where *A* and *B* are the auto- and manual-segmented volumes, respectively; and 2) the mean value and standard deviation of surface-to-surface distance (SSD): *MSSD* = *mean*_*c*∈*C*_(*min*_*d*∈*D*_ ‖*c* − *d*‖_2_), *SDSSD* = *std*_*c*∈*C*_(*min*_*d*∈*D*_ ‖*c* − *d*‖_2_), where *c* and *d* are points on the A and B volumes’ surfaces *C* and *D*, respectively. For statistical measurement, we conducted a ROC curve analysis, where sensitivity ($TPR=\frac{TP}{TP+FN}$) and specificity ($SPC=\frac{TN}{TN+FP}$) were calculated with voxel-wised *TP* (true positive), *TN* (true negative), *FN* (false negative), and *FP* (false positive). Here, TP and TN are the number of the auto-delineation method classified tumor voxels and non-tumor voxels that agreed with the ground truth. *FN* is the number of voxels classified as tumor voxels in the ground truth but missed by the auto-delineation, and *FP* is the number of voxels classified as tumor voxels by the auto-delineation but not in the ground truth. We performed the ROC curve analysis by plotting the curve of sensitivity against (1-specificity). We calculated the Areas under the ROC curves (AUC) to quantify the classification performance.

## Results

### Validation on BRATS

The BRATS database includes five different target structures: necrosis (label 1), edema (label 2), non-enhanced tumor (label 3), enhanced tumor (label 4), and background. In addition to the individual labeled structures, BRATS defines the whole tumor region, which consists of four structures (label 1+2+3+4), and the tumor core, which consists of necrosis, enhanced and non-enhanced tumor (label 1+3+4). As described by Menze *et al*. [27], the edema is segmented on T2 and FLAIR images, and the gross tumor is delineated on T1c together with T1 images, while necrosis and enhanced tumor are segmented on T1c images. Non-enhanced tumor is a residual volume derived by subtracting necrosis and enhanced tumor volumes from the gross tumor volume.

#### Multi-modality segmentation–Comparison with methods in BRATS

For fair comparison with the multi-modality-based methods in BRATS, we trained both the DeepMedic architecture and our En-DeepMedic architecture with T1c and FLAIR images. Table 2 lists the mean DC (± standard deviation, SD) for each method. Compared with other algorithms in the BRATS2015 challenge, our En-DeepMedic method achieved better DCs than most of the reported methods (Table 2).

**Table 2:** Performance of DCs (±SD) in BRATS challenge 2015.

METHOD	Tumor core	Enhanced tumor
**Brain Tumor Segmentation by a Generative Model with a Prior on Tumor Shape[29]**	0.64±0.29	0.52±0.33
**Segmentation of Gliomas in Multi-modal Magnetic Resonance Imaging Volumes Based on a Hybrid Generative-Discriminative Framework[30]**	0.77	0.68
**Multi-modal Brain Tumor Segmentation (BRATS) using Sparse Coding and 2-layer Neural Network**^**1**^**[31]**	0.64	-----^2^
**CaBS: A Cascaded Brain Tumor Segmentation Approach[32]**	0.67	0.68
**Parameter Learning for CRF-based Tissue Segmentation of Brain Tumors**^**3**^**[33]**	(0.78, 0.91)	(0.81,0.92)
**Deep Convolutional Neural Networks for the Segmentation of Gliomas in Multi-Sequence MRI [34]**	0.73	0.68
**Multi-Modal Brain Tumor Segmentation Using Stacked Denoising Autoencoders [35]**	0.72±0.17	0.66±0.18
**DeepMedic [22]**	0.75	0.72
**En-DeepMedic method**	0.75±0.07	0.81±0.04

^1^The authors declared their DCs performance in median value

^2^The authors didn’t provide corresponding data

^3^The authors declared their DCs performance in format (median, range = max-min)

#### T1c segmentation–Comparison with DeepMedic

**Fig 5:**
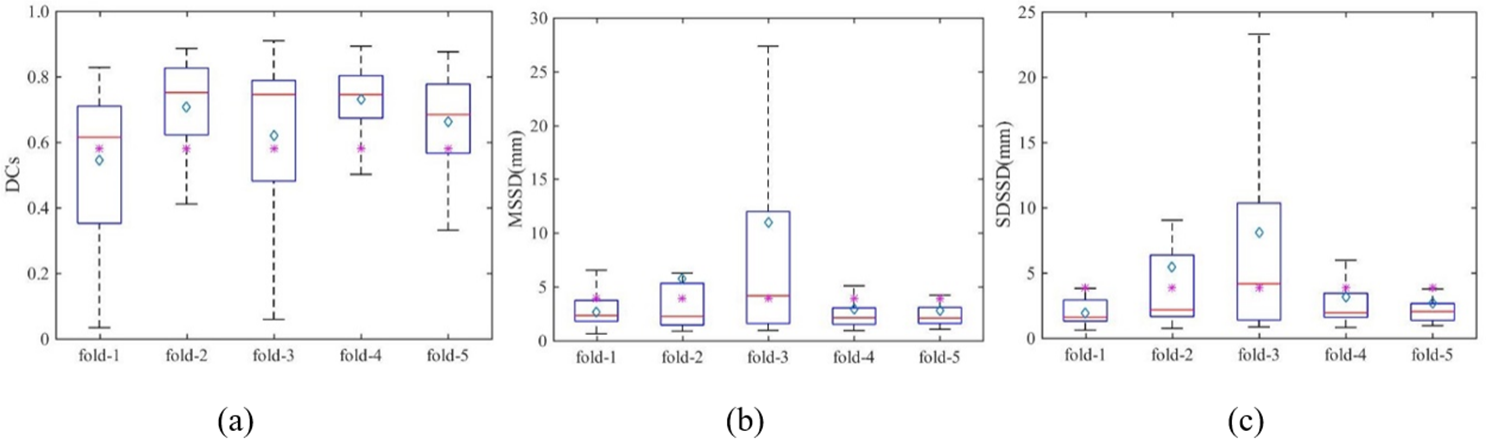
Box plots of geometrical metrics. (a) DCs, (b) MSSD, and (c) SDSSD of 5-fold cross validation with BRATS data using En-DeepMedic. The red line and cyan diamond inside each box denote medium value and mean value, respectively. The magenta star indicates the mean value of the geometrical metrics results from DeepMedic.

Since our method is primarily intended for mono-modality segmentation, we trained the model using only the T1c image with a 5-fold cross validation. The quantitative results given by geometrical metrics are shown in Fig 5. The mean value of each metric resulting from DeepMedic is also plotted using magenta stars in the figure for the comparison.

**Fig 6:**
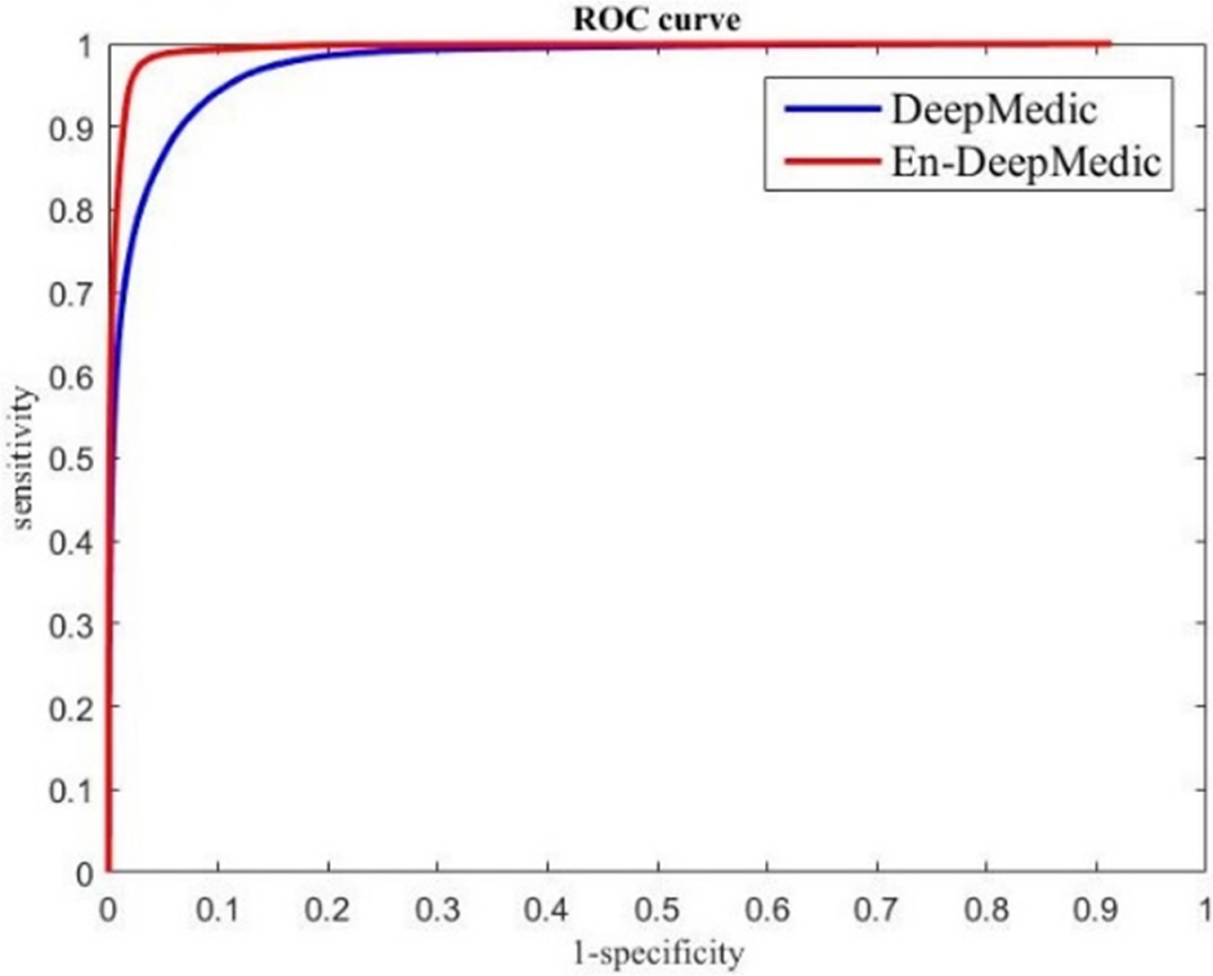
ROC curves for using DeepMedic and En-DeepMedic methods for BRATS data segmentation.

The ROC curves measuring the classification performance are plotted in Fig 6. The red curve indicates the developed En-DeepMedic model, and the blue curve indicates the DeepMedic model. We calculated the AUC of the two curves and obtained 0.99 for the developed En-DeepMedic method and 0.97 for DeepMedic.

### Validation with clinical data

#### Sample cases

**Fig 7:**
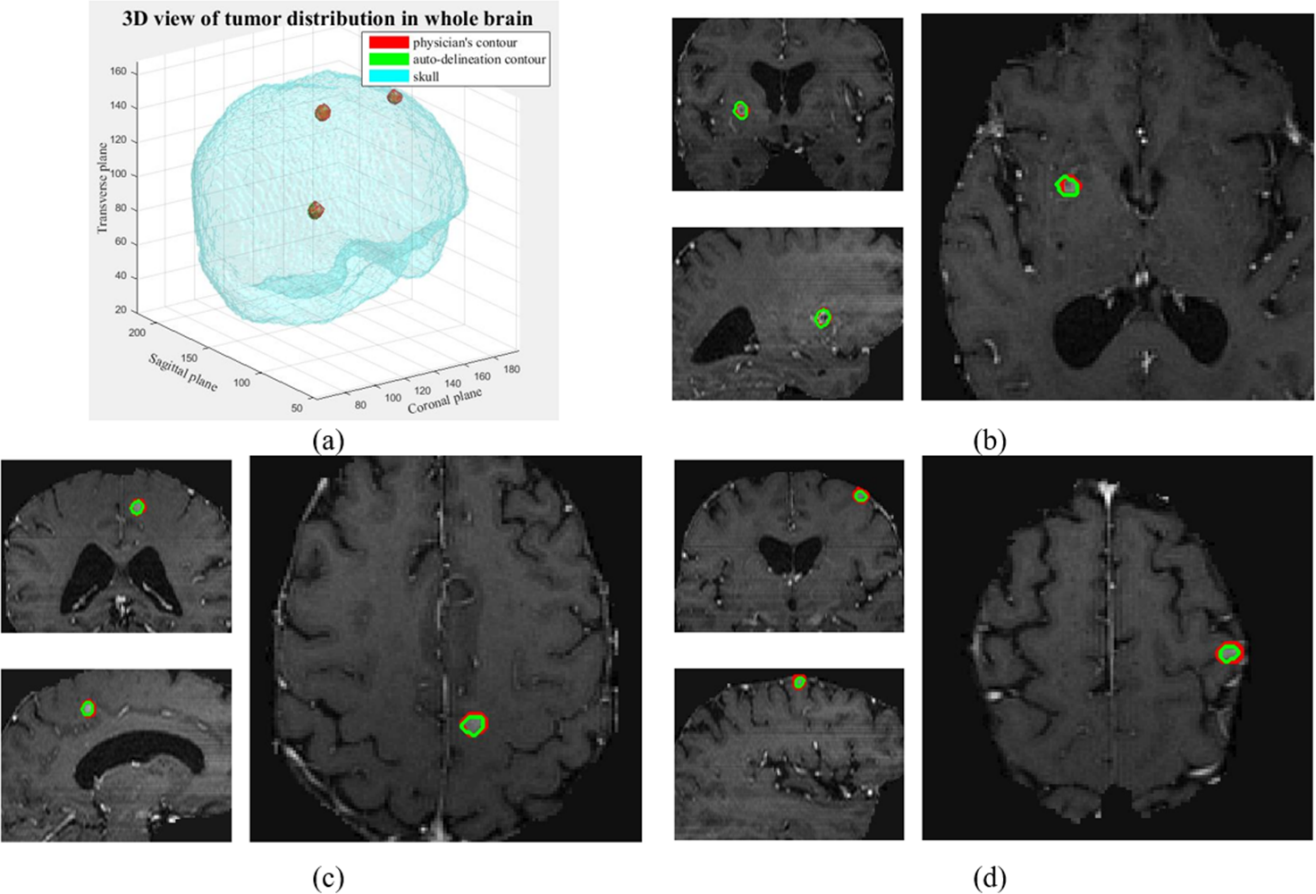
Segmentation results of Pt #1. (a) 3D rendering of the tumor locations inside the brain; (b)~(d): segmentation of lesion in (b) the right globus pallidus, (c) the left paracentral lobule, and (d) left mid frontal.

**Fig 8:**
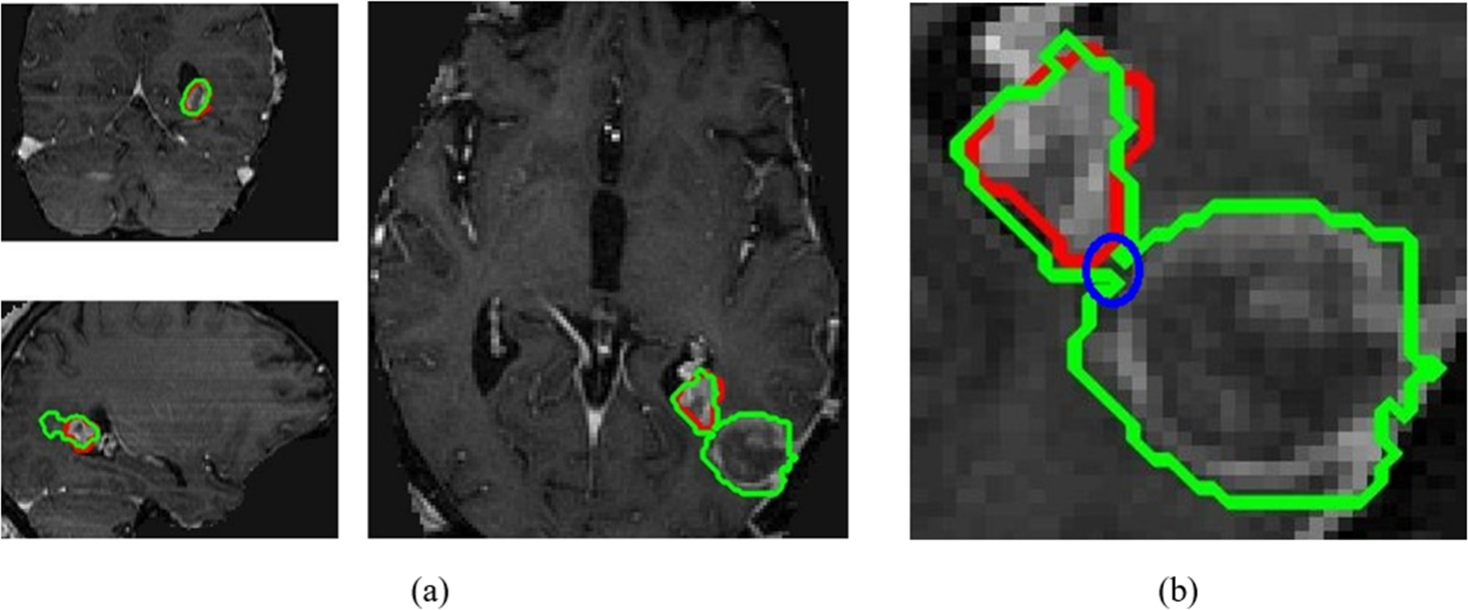
Segmentation results of Pt #2. (a) Segmentation of lesion in the left choroid plexus; (b): zoomed-in of the transverse view in (a).

We present two sample cases to illustrate En-DeepMedic performance on small tumor segmentation. The first case (named Pt. #1) is a 65-year-old male patient who underwent Gamma Knife radiosurgery for brain metastasis of melanoma. There were three lesions, including a 0.221cc volume in the left mid frontal, a 0.276cc volume lesion in the left paracentral lobule, and a 0.293cc volume in the right globus pallidus, respectively. The distribution of the lesions is shown in Fig 7(A), where the red contours represent the ground truth, and the green contours illustrate the result of the En-DeepMedic method. Fig 7(B), 7(C) and 7(D) show three lesions’ delineation results, each with coronal, sagittal, and transverse views. The auto-delineated contours overlap with the manual ones and achieve an average DC of 0.84. The second case (named Pt. #2) is a 47-year-old female patient who underwent Gamma Knife radiosurgery for brain metastasis of melanoma. This patient has one lesion located in the left choroid plexus with size of 0.194cc and another previous treated lesion in the post temporal lobe. The delineation results on 3 orthogonal cross sections views are shown in Fig 8. The quantitative evaluation of these two patients segmentation results are listed in Table 3.

**Table 3:** Quantitative geometrical accuracy of each lesion of sample cases.

Patient #	Lesion position	DCs	MSSD(mm)	SDSSD(mm)
**Pt. #1**	right globus pallidus	0.87	0.5	0.5
	left paracentral lobule	0.85	0.4	0.5
	left mid frontal	0.79	0.5	0.5
**Pt. #2**	left choroid plexus	0.20	12.6	8.8

#### Overall performance

**Fig 9:**
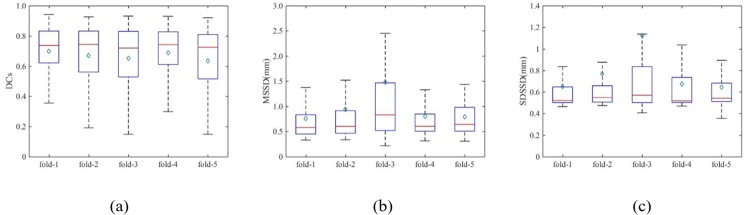
Box plots of clinical patient data 5-fold cross validation results. (a) DCs, (b) MSSD, and (c) SDSSD using the En-DeepMedic method. The red line and the cyan diamond inside each box denote medium value and mean value, respectively.

**Fig 10:**
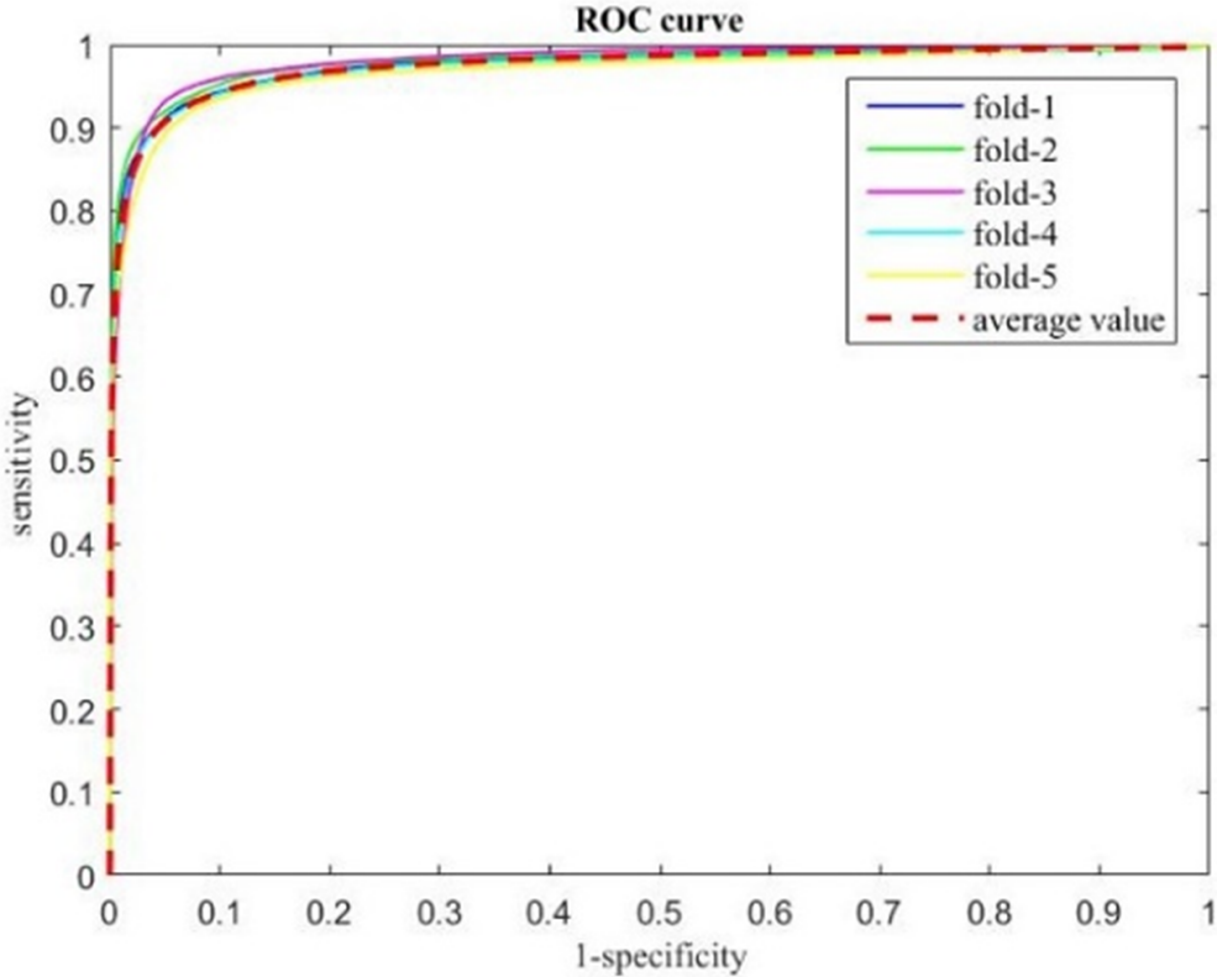
ROC curves of using En-DeepMedic for patient data segmentation.

We employed a 5-fold cross validation strategy to estimate the overall performance of the segmentation strategy. The resulting mean values for the geometrical metrics are: DCs 0.67±0.03, MSSD 0.9±0.3mm, and SDSSD 0.8±0.1mm. The detailed distributions of each geometrical metric are shown in Fig 9. The ROC curves of the En-DeepMedic classifier from each fold are plotted in Fig 10 by the five colored solid lines, and the red dotted line indicates the average curve. AUC is 0.98±0.01, close to 1.

### Comparison with our previous intensity-based segmentation method

**Fig 11:**
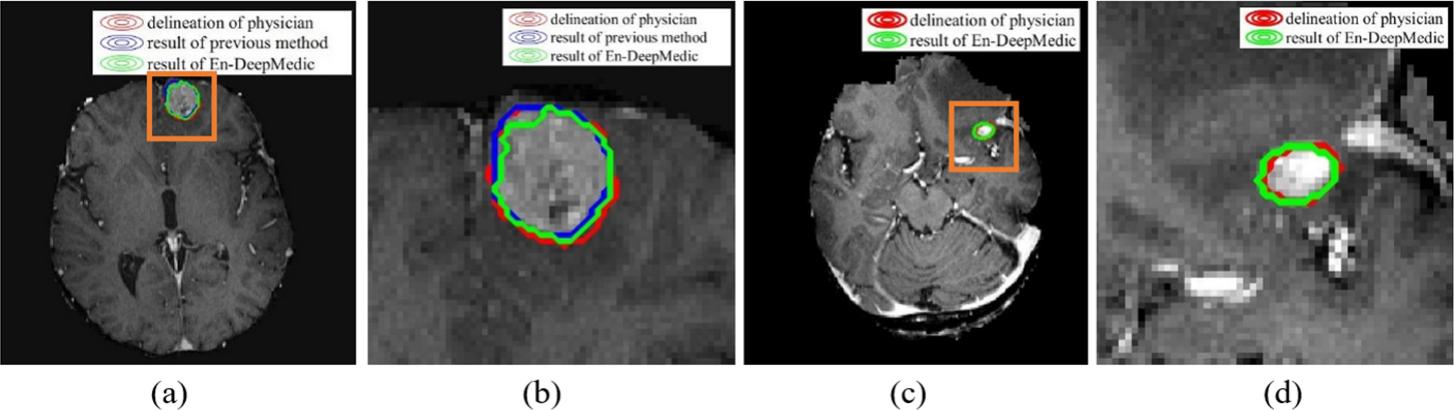
Segmentation results. (a) the large tumor (2.532cc), auto-contours from intensity-based method (blue) and En-DeepMedic method (green) overlaid on physician drawn contour (red); (b) zoomed-in view of ROI in orange rectangle in (a); (c) the small tumor (0.537cc), auto-contours from En-DeepMedic method (green) overlaid on physician drawn contour (red); (d) zoomed-in view of ROI in orange rectangle in (c).

We compared the En-DeepMedic method with our previous intensity-based method [7]. We selected ten tumors with different sizes, ranging from 6.918cc to 0.129cc. Both methods delineated the large lesions (≥1.500cc) successfully. Fig 11 illustrates the delineation results of a large tumor (2.532cc) and a small tumor (0.537cc). Table 4 lists the corresponding geometrical metrics values from both methods. The small lesions were more challenging, and the intensity-based method failed to delineate 4 of the 6 metastases smaller than 1.500cc. In contrast, the En-DeepMedic performed well with the small lesions.

**Table 4:** Comparison geometrical metric values of En-DeepMedic and intensity-based method on small- and large-size brain metastases tumors.

Tumor (cc)	DCs		MSSD(mm)		SDSSD(mm)	
	En-DeepMedic method	Intensity-based method[7]	En-DeepMedic method	Intensity-based method[7]	En-DeepMedic method	Intensity-based method[7]
**6.918**	0.93	0.93	0.6	0.7	0.6	1.4
**3.928**	0.93	0.34	0.5	2.5	0.6	1.5
**2.532**	0.89	0.94	0.8	0.4	0.9	0.5
**1.476**	0.89	0.94	0.7	0.4	0.7	0.5
**0.825**	0.86	-----^1^	0.5	-----	0.5	-----
**0.537**	0.88	-----	0.5	-----	0.5	-----
**0.326**	0.90	-----	0.4	-----	0.5	-----
**0.293**	0.87	-----	0.5	-----	0.5	-----
**0.163**	0.80	0.80	0.3	0.4	0.5	0.5
**0.129**	0.87	0.74	0.4	0.7	0.5	0.5

^1^---indicates there are no values associated with the intensity-based method, since the intensity-based method is not able to detect tumor.

## Discussion

In this study, we presented En-DeepMedic, a CNN-based delineation method designed for automatic brain metastases segmentation for radiosurgery. To compare with other algorithms, the developed En-DeepMedic method was trained on multi-modality BRATS data and achieved good DCs. The competitive results obtained from BRATS2015 are shown in Table 2. The developed method ranked first in the enhanced tumor region (label 4 in BRATs) and second in the tumor core (label 1+3+4). This result indicates that En-DeepMedic performs well on multimodality image segmentation. Also, our En-DeepMedic is mainly designed for delineating small lesions. It performs well on BRAST2015 dataset which accurately doesn’t have much small size brain tumors. We believe if we adjust some hyper-parameters and image patch size, the En-DeepMedic could achieve even better performance. Our En-DeepMedic also performed better than its parent DeepMedic method on mono-modality segmentation (Figs 5 and 6). We achieved this improvement by adding an additional sub-path (sub-path 2) to the architecture, as shown in Fig 2. Sub-path 2 employed larger convolution filters (5,5,5) compared to the filter size (3,3,3) in sub-path 1, and thus captures different image features than sub-path 1. The hybrid features learned from sub-paths of different filter sizes and multiple scales can provide comprehensive local and global information for accurate classification of voxels. Our En-DeepMedic method also performed well in evaluating clinical data with satisfactory geometric accuracy and ROC curve analysis (Figs 9 and 10). The achieved AUC was 0.98±0.01, which indicates good performance.

The sample case shown in Fig 8 didn’t achieve high DC with En-DeepMedic method, even though the location of lesion was correctly identified. The main reason for this low DC is that two contours are merged together. The targeted lesion in the left choroid plexus is close to the abnormality in the post temporal lobe that was treated 5 months ago. The shortest separation between two lesions boundaries is ~ 1mm, which is the size about or smaller than the voxel resolution as highlighted by the blue circle in Fig 8(B). Our En-DeepMedic delineation method was failed to separate them. To overcome this problem, contour post-processing is needed.

Compared to our previous intensity-based method, the En-DeepMedic method achieved superior results in small tumor segmentation, while the previous method often fails to detect tumors smaller than 1.500cc. We chose 10 cases that varied from 6.918cc to 0.129cc and used both methods to conduct segmentation. Both methods succeeded in auto-segmenting lesions larger than 1.500cc, but the intensity-based method failed on four of the six small cases, while the En-DeepMedic method worked robustly (Table 4). The two methods are, however, designed for different applications [7]. The intensity-based method can capture abnormalities without any prior information; the only input is the image set’s intensity. Generally, this method requires larger tumor volumes that have adequate voxels representing discriminative information. This constraint limits its application for small lesion segmentation. The En-DeepMedic method is better suited to small lesion segmentation, as it employs a deep learning strategy and classifies each voxel individually based on knowledge learned from a training dataset. This method considers multi-scale information and the nonlinear relationship between neighboring voxels. Theoretically, this voxel-wise segmentation strategy has the capability to segment a tumor, even if it is formed by only one voxel. However, the En-DeepMedic method results in a relatively zigzag contour when delineating large targets compared to our previous intensity-based method (Fig 11(B)), because the voxel-wise classification strategy cannot guarantee a smooth contour, while our previous method includes a contour evolving step that smoothes contours automatically. To overcome this limitation, additional postprocessing methods, such as using a localized active contour model [36], could be employed to refine the final contours, but the postprocessing step would increase computation time.

**Fig 12:**
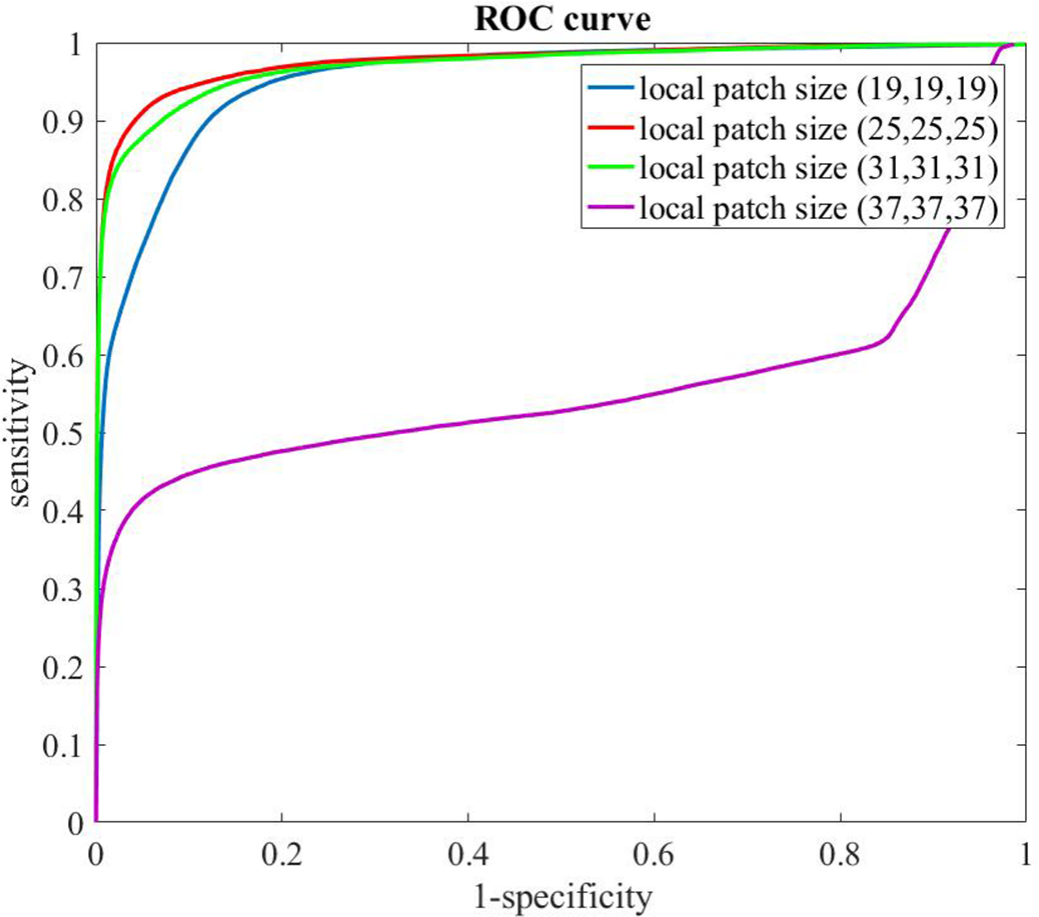
ROC curve of the En-DeepMedic with different local patch size.

The input image patch size affects the performance of En-DeepMedic algorithm. A large image patch size requires a big memory allocated on the GPU. Our study is conducted on a laptop with an NVIDIA Quadro M2000M graphic card and an Intel Xeon E3-1505 processor. The largest local image patches the computer can handle is (37,37,37). We evaluate the En-DeepMedic performance with local patch sizes varied from (19,19,19) to (37,37,37) on our clinical dataset. One thing we have to point out is that some hyper parameters (training, validating, and testing batch size) are revised to fit GPU computation resource limit. For the local patch size (19,19,19) and (25,25,25), batch sizes are the same as those given in Table 1. For the local patch size (31,31,31), the batch size for training, validation and testing are 5,9, 10, respectively. For the local patch size (37,37,37), the batch size for training, validation and testing are revised as 1, 9 and 10, respectively. The results of quantitative geometry metrics are listed in Table 5 and ROC curve are plotted in Fig 12. As results shown, the best The best geometric metric and ROC are both achieved at a local patch size of (25,25,25).

**Table 5:** The quantitative metrics of the En-DeepMedic with different local patch size.

Local patch size	DCs	MSSD(mm)	STSSD(mm)
**(19,19,19)**	0.52 ± 0.25	1.4 ± 0.9	1.1 ± 0.7
**(25,25,25)**	0.70 ± 0.19	0.8 ± 0.6	0.7 ± 0.3
**(31,31,31)**	0.60 ± 0.23	0.9 ± 0.4	0.7 ± 0.2
**(37,37,37)**	0.52 ± 0.28	0.9 ± 0.4	0.7 ± 0.3

Based on the results above, we choose the local patch size (25,25,25), the corresponding global patch size is (57,57,57), and the down-sampled global patch size is (19,19,19).

Another advantage of the En-DeepMedic method is computational efficiency. Though it takes time to train a CNN model, segmentation itself is fast. In our study, the entire workflow was implemented using a laptop with an NVIDIA Quadro M2000M graphic card and an Intel Xeon E3-1505 processor. The training of each fold of clinical data takes about two days, but auto-segmenting tumors on a typical image set with the size of 256 × 256 × 176 takes approximately two minutes. Moreover, the segmentation time is independent of the number of tumors, since it classifies voxels in an image all at once.

The En-DeepMedic method is also image modality-independent. It can be applied to both multi-modality and mono-modality images, as long as the training data used are the same modality as the images to be segmented. Since our application focuses on SRS of brain metastases, we thoroughly evaluated its performance on mono-modality T1c image data. However, the training and testing results on multi-modality BRATS data already show its promise for expanding to multi-modality image segmentation. Another important feature of our En-DeepMedic method is that it is parameter-less. No parameters need tuning after the model is established, so the segmentation is more robust and requires less human intervention.

## Conclusion

In this work, we developed a deep convolutional neural network machine learning method for automatic segmentation of small brain metastases. We validated the method comprehensively on both BRATS and clinical data sets, and it demonstrated superior performance when compared with reported algorithms in the BRATS challenge. The developed auto-segmentation strategy achieved high accuracy and efficiency and shows promise as a tool for accurate and efficient SRS treatment planning.
